# A Novel Graphic-Aided Algorithm (gNIPT) Improves the Accuracy of Noninvasive Prenatal Testing

**DOI:** 10.1155/2020/4712657

**Published:** 2020-07-25

**Authors:** Qingwen Zhu, Jing Wang, Xiaoning Xu, Shiying Zhou, Zhengli Liao, Jun Zhang, Lingyin Kong, Bo Liang, Xiaoyan Cheng

**Affiliations:** ^1^Prenatal Screening and Diagnosis Center, Nantong Municipal Maternal and Child Health Hospital, Nantong, 226010 Jiangsu, China; ^2^Basecare Medical Device Co., Ltd., Suzhou, Jiangsu, China; ^3^State Key Laboratory of Microbial Metabolism, Joint International Research Laboratory of Metabolic and Developmental Sciences, School of Life Sciences and Biotechnology, Shanghai Jiao Tong University, Shanghai, China

## Abstract

Noninvasive Prenatal Testing (NIPT) has advanced the detection of fetal chromosomal aneuploidy by analyzing cell-free DNA in peripheral maternal blood. The statistic *Z*-test that it utilizes, which measures the deviation of each chromosome dosage from its negative control, is now widely accepted in clinical practice. However, when a chromosome has loss and gain regions which offset each other in the *z*-score calculation, merely using the *Z*-test for the result tends to be erroneous. To improve the performance of NIPT in this aspect, a novel graphic-aided algorithm (gNIPT) that requires no extra experiment procedures is reported in this study. In addition to the *Z*-test, this method provides a detailed analysis of each chromosome by dividing each chromosome into multiple 2 Mb size windows, calculating the *z*-score and copy number variation of each window, and visualizing the *z*-scores for each chromosome in a line chart. Data from 13537 singleton pregnancy women were analyzed and compared using both the normal NIPT (nNIPT) analysis and the gNIPT method. The gNIPT method had significantly improved the overall positive predictive value (PPV) of nNIPT (88.14% vs. 68.00%, *p* = 0.0041) and the PPV for trisomy 21 (T21) detection (93.02% vs. 71.43%, *p* = 0.0037). There were no significant differences between gNIPT and nNIPT in PPV for trisomy 18 (T18) detection (88.89% vs. 63.64%, *p* = 0.1974) and in PPV for trisomy 13 (T13) detection (57.14% vs. 50.00%, *p* = 0.8004). One false-negative T18 case in nNIPT was detected by gNIPT, which demonstrates the potency of gNIPT in discerning chromosomes that have variation in multiple regions with an offsetting effect in *z*-score calculation. The gNIPT was also able to detect copy number variation (CNV) in chromosomes, and one case with pathogenic CNV was detected during the study. With no additional test requirement, gNIPT presents a reasonable solution in improving the accuracy of normal NIPT.

## 1. Introduction

Since the discovery of the presence of cell-free fetal DNA (cffDNA) in maternal peripheral blood in 1997 [1], various strategies have been proposed to develop screening or diagnostic tests for fetal aneuploidy. In 2008, two studies sequenced maternal cfDNA with massively parallel sequencing to assess the trisomy of chromosome 21 for Down's syndrome [2, 3]. Extended to include detection of abnormity in chromosome 18 and chromosome 13 [4], which corresponds to Edward's syndrome and Patau's syndrome, respectively, this method of directly detecting fetal chromosomal abnormalities is widely known as NIPT [5]. The noninvasive nature of NIPT has its strength in avoiding the unnecessary risk of fetal miscarriage, comparing to conventional prenatal diagnosis, such as amniocentesis, which is the golden standard of prenatal diagnosis for fetal chromosomal abnormalities and involves the invasive sampling of fetal materials [6]. Other noninvasive methods, such as serological test or ultrasonography, have a high false-positive rate and false-negative rate. As NIPT has become sufficiently robust for pregnant woman at high risk for fetal aneuploidy, several committees, including the American College of Medical Genetics and Genomics [7] and the National Society of Genetic Counselors [8], had published positions for cffDNA testing for high-risk pregnant women after counseling.

The statistical *Z*-test of unique read count analysis was utilized as a significant difference test [2] to overcome the challenge that fetal DNA only represents a small proportion of the maternal plasma with the majority of the plasma being maternal DNA in NIPT [9]. It measures the number of standard deviations of the sample chromosome from the mean of a negative reference dataset. Expanded to the detection from T21 to T18 and T13 detection, the *Z*-test would often be preceded with read correction with GC content to eliminate the effect of GC bias on sequenced read counts [4]. Depending on the reference, there can be a variety of *Z*-tests [3, 10]. The vast majority of NIPT products for fetal chromosome aneuploidy detection on the market are based on this algorithm [11–13].

The *Z*-test treats each chromosome as a whole and calculates uniquely mapped reads to this whole chromosome. It may bring errors in the result when regions of a chromosome have an offsetting effect during *z*-score calculation. If one or several regions had copy-number loss while another or other regions of the same chromosome had copy-number gain and these two variations rendered an offsetting effect during *z*-score calculation, the nNIPT would not be able to detect such cases. In the case of false-negative results, which brings a great burden to the related family as well as society, it would be of great necessity to avoid such cases even at a price of a slightly higher false-positive rate.

In this paper, gNIPT is demonstrated to significantly improve the PPV of the overall NIPT test and especially for T21. In this gNIPT, the widely used *z*-score calculation in NIPT was complemented with *z*-scores for each 2 Mb window of each chromosome and each window's copy number variation measured with *z*-score. Overall, gNIPT significantly improved the overall PPV from 66.67% to 88.14% (*p* = 0.0041). For T21 detection, both methods had equal results in sensitivity, specificity, and negative predictive value (NPV), with the PPV significantly improved from 71.43% to 93.02% (*p* = 0.0037). Though not statistically significant, there was also improvement in the detection of T18 and T13. The correlation analysis between gNIPT and nNIPT showed a strong correlation (Cohen's Kappa coefficient 0.84), which shows that the gNIPT would agree with the nNIPT result in most test cases. In addition, gNIPT could also identify false-negative cases and detect CNVs without extra requirement. This gNIPT method has great potential in improving the accuracy of nNIPT.

## 2. Materials and Methods

### 2.1. Sample Collection

During the three years' period from January 13, 2016, to May 11, 2019, 13537 pregnant women who meet the requirement of the NIPT technical specifications in the Prenatal Screening and Diagnosis Center, Nantong Municipal Maternal and Child Health Hospital, Jiangsu Province, were enrolled in the NIPT test. The institutional review board of the hospital approved the test. Written informed consent from all participants was obtained before the test. The maternal ages for the 13537 pregnancies were within the range of 16 to 48 years old, and all cases were singleton pregnancies. The gestational age ranged from 12 to 36 weeks. The basic information about the distribution of maternal age and gestational age is presented in Table 1.

### 2.2. Library Construction

A two-step centrifugation process was performed to extract plasma from 10 ml whole blood samples of pregnant women: (1) tubes of blood were centrifuged at 1,600 × g for 10 min at 4°C, and the plasma was then transferred to microcentrifuge tubes; (2) the plasma in the microcentrifuge tubes were centrifuged at 16,000 × g for 10 min to remove residual cells and obtain cell-free plasma. The cell-free plasma was stored at -80°C before DNA extraction. DNA fragments were extracted from 600 *μ*l cell-free plasma using the Circulating Nucleic Acid Kit (Qiagen, Germany). An Ion Plus Fragment Library Kit (Life Technologies, USA) for the Ion Proton platform was used to construct the sequencing library for each plasma sample, and the libraries were quantified on a Qubit Fluorometer (Thermo Fisher Scientific, USA).

### 2.3. Sequencing

The sequencing library was loaded onto an Ion P2 chip. A standard 160 cycle of Ion Torrent sequencing was run in a single-end sequencing model. The primary sequencing data were processed by the Ion Torrent platform-specific pipeline software (Torrent Suite, version 4.4.3) in order to generate sequence reads, to trim adapter sequences, and to filter out low-quality reads.

### 2.4. Data Analysis

The sequencing data after adapter trimming and quality filtering were mapped to the hg19 human reference genome (version: NCBI Build37/hg19) by bowtie2 software [14]. Four types of mapped reads: PCR duplicates, short reads (shorter than 35 bp), multimapped reads, and low-quality reads (MAPQ score < 60), were removed. The Ion Torrent platform-specific pipeline software (Torrent Suite, version 4.4.3) was used for data preprocessing [15]. A minimal coverage of uniquely mapped reads requirement is 0.16-fold (0.16 X) for both nNIPT and gNIPT. The data analysis standard and aneuploidy criteria were based on studies reported previously, which demonstrated that an optimized algorithm in massively parallel sequencing was capable of detecting multiple fetal chromosomal abnormalities [16, 17].

#### 2.4.1. nNIPT

Sequences that could only map to one location of the chromosomes were counted as uniquely mapped reads (UMRs). The percentage of reads mapped to each chromosome was calculated using the number of UMRs in a selected chromosome divided by the count of UMRs in all chromosomes after normalizing the number of UMRs by LOESS regression for executing GC correction [4]. To determine the disease status of the chromosome, a reference dataset in which all samples were obtained from maternal plasma of euploid pregnancies was used as the baseline for the calculation of *z*-score [2]. The aneuploidy state of the interested chromosome was classified based on the statistical significance of the *z*-score. An absolute value of *z*-score, 3.00, was used as the standard. A chromosome with an absolute value of *z*-score more than 3.00 or equal to 3.00 was classified as the affected chromosome, and a chromosome with the absolute value of *z*-score less than 3.00 was classified as an unaffected chromosome.

#### 2.4.2. gNIPT

In the nNIPT analysis, the UMR-based *Z*-score calculation treats the whole chromosome as one unit. It may conceal reads imbalance cases in which one region has an extremely large number of reads while another region's number of reads is especially small and making such case to appear normal. gNIPT is able to detect such kind of abnormality. The gNIPT was enabled by complementing nNIPT with detailed chromosome analysis by dividing each chromosome into 2 Mb size windows and treating each window as a single unit. Each window's *z*-score was calculated based on the 2 Mb segment of the reference dataset, with the following equation (chrN represents the chromosome of interest):

(1) $$\begin{array}{c} \text{chrN }2\text{ Mb }z\text{ score}=\frac{\%\text{chrN }2\text{ M}{\text{b}}_{\text{sample}}-\text{mean}\%\text{chrN }2\text{ M}{\text{b}}_{\text{reference}}}{\text{SD}\%\text{chrN }2\text{ M}{\text{b}}_{\text{reference}}}. \end{array}$$

The percentage of UMRs of the 2 Mb window of the sample was compared with the same chromosome region of the reference dataset. The deviation of the interested window from the reference data was also obtained with a *Z*-test. In the line chart, the calculated *z*-scores for each chromosome were plotted and ligated with a green line to directly reveal the status of each window. If the absolute value of *z*-score equals or exceeds 3, a filled black dot would be used to indicate potential abnormity. In a normal condition, the green line would fluctuate around the *x*-axis. Additionally, the *z*-scores of each chromosome were passed as a one-dimensional vector into the R package cghFlasso (version 0.2.1) for CNV analysis and the result would be plotted with a purple line. If several consecutive windows have *z*-scores larger than zero, resulting in a segment of the line above the *x*-axis, the cghFlasso algorithm would classify the region as a block and calculate the *z* value for the block with the weighted *z*-score of each window. If the absolute value of *z*-score equals or exceeds 3.00, the window would be marked with a filled red dot in the purple line to indicate potential abnormity in CNV.

The calculation method of the *z*-score in the gNIPT is the same as in the nNIPT. A chromosome with an absolute value of *z*-score more than 3.00 or equal to 3.00 was classified as the affected chromosome, and a chromosome with an absolute value of *z*-score less than 3.00 was classified as an unaffected chromosome. To determine the disease status of the chromosome, both the results from the line chart and the *z*-score are considered.

#### 2.4.3. Verification of NIPT Results

The pregnant women with a high risk in nNIPT and gNIPT results were further examined by amniocentesis. A 20 ml sample of amniotic fluid or a 1 ml sample of umbilical cord blood from each case was extracted for cell culture and chromosome karyotype analysis. A SNP microarray analysis on the Affymetrix platform (Santa Clara, California, USA) would be performed later for verification. For pregnant women with high-risk NIPT results, chromosome karyotype analysis or Chromosome Microarray Analysis (CMA) and follow-up visits were performed for verification. In cases where amniocentesis was not performed, umbilical cord blood was taken at delivery for karyotyping. Other low-risk NIPT cases were validated by telephone follow-ups.

#### 2.4.4. Statistical Analysis

Statistical analyses were performed using the R statistical package (version 3.6.1; R Foundation for Statistical Computing, Vienna, Austria). Welch's unequal variances *t*-test was applied to calculate the statistical difference between gNIPT and nNIPT. Cohen's Kappa coefficient [18] was calculated for the analysis of the coincidence rate of the gNIPT and nNIPT. The statistical *Z*-test was used to identify fetal aneuploidies in the nNIPT, and both *Z*-test and graphics were used to identify fetal aneuploidies in the gNIPT.

## 3. Results

### 3.1. gNIPT Improves the Accuracy of nNIPT

From January 13, 2016, to May 11, 2019, 13537 singleton pregnancy women participated in this study. The demographic characteristics of the participants are shown in Table 1. The median maternal age of the population was 29 years (ranged from 16 to 48 years). The majority of maternal age was within the 26 to 30 years range, taking up about 40.4% of the population. About 14.74% (1995/13537) of pregnant women had an advanced maternal age of 36 years or more. NIPT was performed at a gestational age of 12–36 weeks, with an average of 18.2 weeks, and mainly at 16–19 weeks (78.05%).

**Figure 1:**
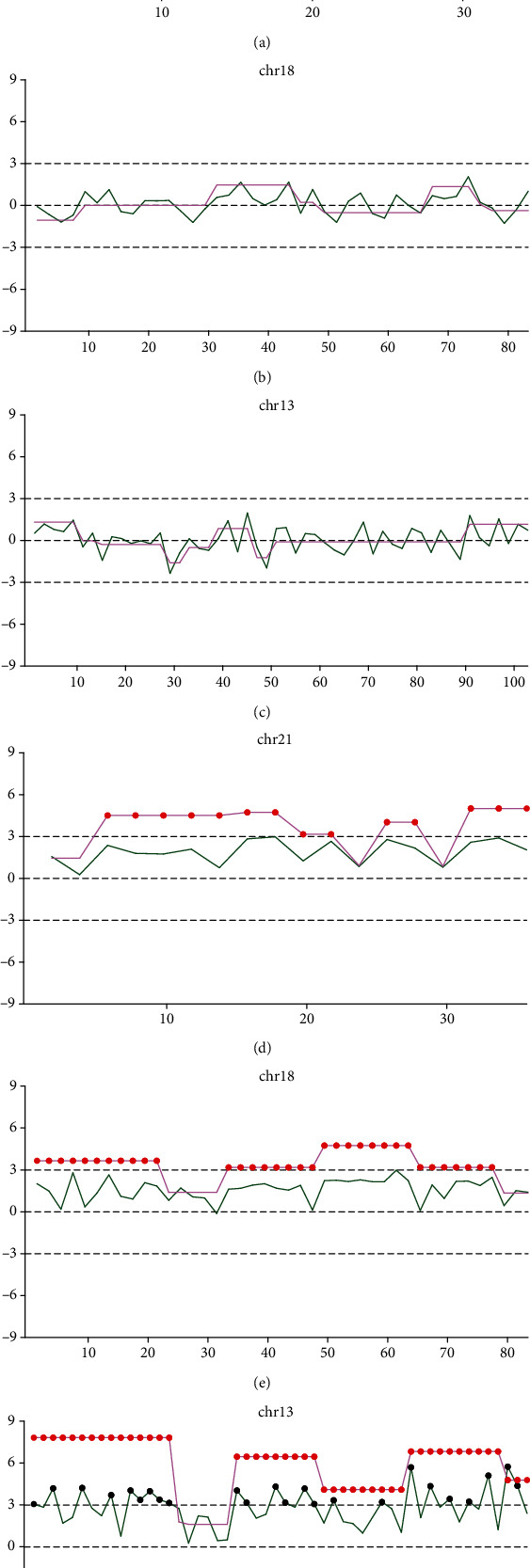
Classic graph for both positive and negative cases of chr21, chr18, and chr13 in gNIPT analysis. (a) Normal chromosome 21 (negative case). (b) Normal chromosome 18 (negative case). (c) Normal chromosome 13 (negative case). (d) Trisomy 21 (positive case). (e) Trisomy 18 (positive case). (f) Trisomy 13 (positive case).

In the gNIPT analysis, a line chart was utilized to show the status of the chromosome across each 2 Mb size windows, with a green line depicting each 2 Mb size window's *z*-score and a purple line demonstrating the *z*-score of CNV variation for each window. Under normal conditions, the green line and the purple line would fluctuate around the *x*-axis, with almost no parts exceeding the 3 and -3 thresholds, as delineated by Figures 1(a)–1(c). Situations where the green line or purple line passes through each threshold would indicate abnormity in that region of the chromosome. The system would give out warning under two conditions: if five consecutive bins with ∣*Z* | ≥5, indicating an unknown anomaly of a ≥10 Mb region; if two or more consecutive bins with ∣*Z* | ≥5 and involved in a known syndrome, indicating a known anomaly in a region with no less than 4 Mb. For a typical trisomy result, the green line of the chart would have large regions of windows deviating from the *x*-axis, and the purple line would have several consecutive red dots, as depicted in Figures 1(a)–1(f).

In this three years' study, all pregnancies were analyzed with both nNIPT and gNIPT. Of the 13537 samples in which SNP microarray analysis, chromosome karyotype analysis, or follow-up visit were performed for verification, 53 (0.39%, 53/13537) were positive, including 41 for T21, 8 for T18, and 4 for T13. The overall sensitivity and specificity for nNIPT were 96.23% and 99.82%, respectively, as shown in Table 2. Both sensitivity and specificity with the gNIPT method showed a slight increase, the values are 98.11% and 99.95%, respectively. The PPV for the overall test was significantly increased from 66.67% in the nNIPT to 88.14% with the gNIPT test (*p* = 0.0041). For T21 detection, gNIPT had increased the PPV from 71.43% to 93.02%, and this increase is statistically significant (*p* = 0.0037); other results for T21 were similar in both methods, with 97.56% for sensitivity, around 99.90% for specificity, and 99.99% for NPV. For T18 detection, the PPV from gNIPT was 88.89%, and it was 63.64% with the nNIPT method (*p* = 0.3506). Comparing with nNIPT, gNIPT also had better sensitivity, 100.00% versus 87.50% in nNIPT. For T13 detection, gNIPT still had a better PPV value (57.14%) than nNIPT (50.00%), with other measurements almost equal.

In most cases, gNIPT and nNIPT have consistent results. The coincidence rate of gNIPT and nNIPT is 99.85%, in which consistent results were obtained with both methods for 53 positive cases and 13464 negative cases in the 13537 total participants, as shown in Table 3. Cohen's Kappa coefficient [18] for these two methods is 0.84, which indicates near-perfect agreement between these two methods.

### 3.2. gNIPT Discovers One False-Negative Case

**Figure 2:**
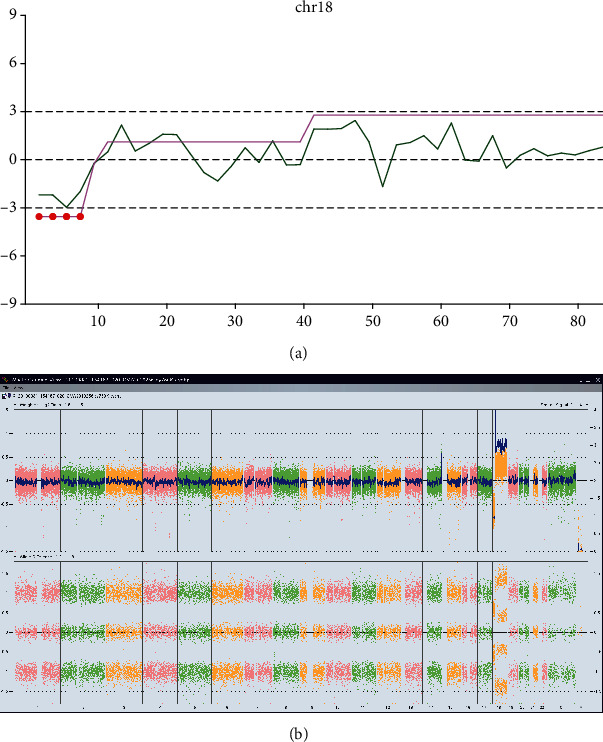
gNIPT analysis and SNP array verification for a T18 false-negative case. (a) Line chart for chr18 showing one region of loss and one region of gain. (b) SNP array verification for the sample, showing abnormal weighted Log2 ratio and allele difference.

A 24-year-old pregnant woman with a gestational age of 17 weeks and 3 days had *z*-scores of 0.477, 1.783, and -1.055 for chr21, chr18, and chr13, respectively, indicating negativity for T21, T18, and T13. However, in the line chart of gNIPT analysis, a segment of the CNV line had *z*-score smaller than -3 with 4 red dots on the purple line, as depicted in the bottom left of Figure 2(a), indicating copy-number loss, and another segment had a value equal to 3, as depicted by the upper right part of Figure 2(a), suggesting copy-number gain of this region. The gNIPT result indicated chr18 abnormity across the whole chromosome.

In the verification process, experiment with SNP array revealed that chr18 had a onefold loss in the region starting from p11.32 to p11.21 (chr18: 136,227-12,675,437) and a threefold gain in the region starting from p11.21 to q23 (chr18: 12,697,649-78,013,728), shown in Figure 2(b). According to the human karyotype map, the two regions with gain and loss cover almost the whole chr18. A consultation to the DatabasE of genomiC varIation and Phenotype in Humans using Ensembl Resources (DECIPHER, https://decipher.sanger.ac.uk) database found that the region with onefold loss encompasses 41 pathogenic records related to global developmental delay and infantile muscular hypotonia and the region gained (chr18 p11.21 to q23) contains 18 similar records. Thus, this pregnancy was determined to have a high risk for T18 and gNIPT had corrected the nNIPT from making a false-negative case.

### 3.3. gNIPT Discovers Pathogenic CNV

**Figure 3:**
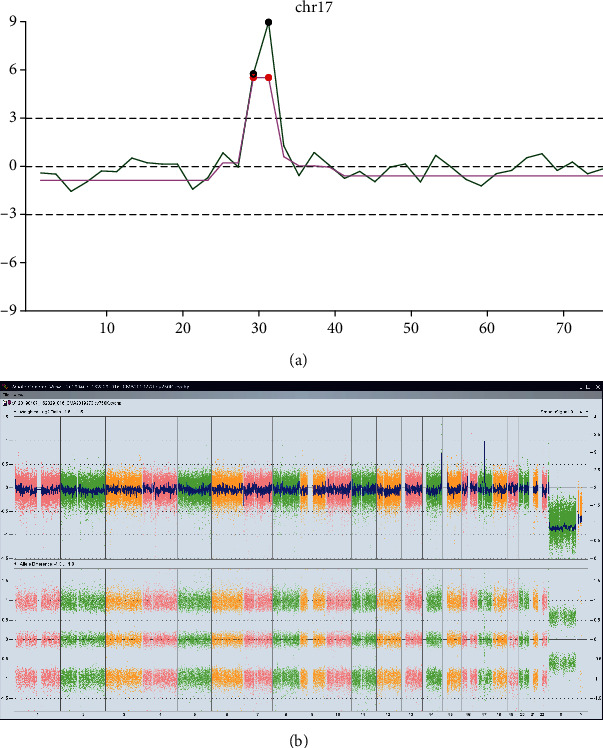
gNIPT analysis and SNP array verification for a chr17 pathogenic CNV. (a) Line chart for chr17 showing one region with 2 black dots on the green line and 2 red dots on the purple line. (b) SNP array verification for the sample, showing abnormal weighted Log2 ratio and allele difference.

In this study, using gNIPT, one pathogenic CNV of chr17 had also been discovered. In the nNIPT, a 36-year-old pregnant woman with a gestational week of 16 had *z*-scores with 1.195, -0.528, and -1.174 for chr21, chr18, and chr13, respectively, indicating negativity for T21, T18, and T13 disease. The *z*-scores for other chromosomes are within the normal range. Though the *z*-score for chr17 was 0.677, which was within the normal range, chr17 of this sample had 2 black dots in the green line and 2 red dots in the purple line of the line chart for gNIPT analysis, as shown in Figure 3(a), indicating copy number gain in this region.

The results from Affymetrix CytoScan 750K SNP Array found a 1868 Kb repeat in 17q12. This region of the chromosome is directly related to 23 Online Mendelian Inheritance in Man (OMIM) genes, among which the CCL3L1 gene is related to HIV susceptibility, the PIGW (610275) gene is related to defects in glycosylphosphatidylinositol biosynthesis (autosomal recessive genetic disease), the ACACA (200350) gene is related to acetyl-coenzyme A carboxylase deficiency (autosomal recessive genetic disease), and the HNF1B (189907) gene is associated with diabetic syndrome (autosomal dominant genetic disease). Decipher and International Standards for Cytogenomic Arrays (ISCA) databases have multiple cases of pathogenicity reports related to this repeated segment, which may lead to developmental and morphological abnormalities such as stunting, mental retardation, microcephaly, short stature, and stunted speech. The ClinGen database shows that the fragment has a triple dose effect, which may cause various clinical manifestations such as developmental delay, mental retardation, behavioral problems, epilepsy, microcephaly, and brain abnormalities, as well as mild facial deformities, kidney abnormalities, and esophageal atresia. Genital abnormalities and cardiac and ocular abnormalities have also been reported [19, 20]. Repeating this fragment will cause 17q12 microrepetition syndrome, and its penetrance is about 21.1% (10.6-39.5%). This segment of CNV was determined to be a pathogenic CNV.

## 4. Discussion

NIPT has been widely used to detect T21, T18, and T13. In nNIPT, a chromosome with an absolute value of *z*-score more than 3.00 or equal to 3.00 was classified as affected, and a chromosome with an absolute value of *z*-score less than 3.00 was classified as unaffected. With this criterion, PPVs for T21, T18, and T13 were 71.43%, 63.64%, and 50.00%, respectively, and the overall PPV was 66.67% in the nNIPT of this study. In this study, a novel method with improved accuracy for NIPT is presented. In this method, detailed chromosome analysis was carried out with the graphic-aided algorithm to improve the accuracy of NIPT calling. The correlation analysis between gNIPT and nNIPT showed a strong correlation (Cohen's Kappa coefficient 0.84), which shows that the graphic-aided algorithm of NIPT would still agree with the NIPT result in most test cases.

The discrepancy between the positive rates in this study and other studies may be explained by the difference in the proportion of advanced maternal age pregnancies. The total number of true-positive cases for T21, T18, and T13 is 53 in our study, and the overall positive rate is 0.39% (53/13537). Koumbaris et al. validated their NIPT using 2033 cell-free DNA samples in 2019 and found 27 positive cases for T13/T18/T21. The overall positive rate of their test is 1.32% (27/2033) [21]. Compared with this value, the positive rate of 0.39% in this study seems quite low. However, Koumbaris et al. failed to present the demographic characteristics of their study population, which is of vital importance in determining the positive rates. A higher proportion of high-risk pregnancies could result in a higher positive rate, due to an increased incidence rate for T13/T18/T21 with increased maternal age [22, 23]. The proportion of high-risk pregnant women only accounts for 14.74% in our study, resulting in a lower incidence rate than the population with a higher percentage of advanced maternal age. As a result of this difference in the distribution of high-risk pregnancies for the data, our overall positive rate could be possibly lower than certain studies.

Meanwhile, the positive rate in this study has closer similarity with several large-scale studies compared with Koumbaris' results. A clinical experience in Mainland China with 146958 samples shows that the positive rates of T13/T18/T21 were 0.045%, 0.15%, and 0.52%, respectively, and the overall positive rate was 0.81% [24]. This result is closer to our overall positive rate, and the discrepancy between this study (0.81%) and our result (0.39%) is understandable taking into the account the demographic difference in the study population: in their study, high-risk pregnant women took up 30.05% of the whole population, while the proportion of high-risk pregnant women only accounts for 14.74% in our data. Recently, an even larger retrospective study of 189809 NIPT samples collected from 28 provincial-leveled administrative units in China revealed that the positive rates of T13/T18/T21 were 0.014%, 0.070%, and 0.276%, respectively, and resulted in an overall positive rate of 0.359% (681/189809) [23]. This overall positive rate is even closer to our result. A possible explanation for this close relationship could be that both the large-scale retrospective study and our study utilizes clinical samples in the real world. And this real-world clinical experience data is composed of a larger proportion of low-risk pregnancies than its high-risk counterpart. As a result, the overall positive rate for studies on this kind of population could be potentially lower than certain studies that do not have such property in the composition of data.

PPV is the proportion of patients with positive results who are correctly diagnosed. The PPVs for T21, T18, and T13 in the nNIPT of this study were 71.43%, 63.64%, and 50.00%, respectively, and those values were calculated as 95%, 82%, and 46%, respectively, in Liang et al.'s work [25, 26]. The study conducted by Liang et al. in 2019 has different characteristics of patient demographics from our study: of the 94085 patients studied, there were 38023 (40.41%) high-risk pregnancies, where maternal age was ≥35 years, while the proportion of high-risk pregnant women only accounts for 14.74% in our study. This larger proportion of high-risk pregnancies in Liang et al.'s study could lead to higher prevalence and finally higher PPVs, because PPV is not intrinsic to the test and it also depends on the prevalence [26]. Generally, a higher prevalence of abnormity would indicate a higher PPV.

Moreover, the PPVs of the nNIPT in our study are consistent with several previous studies. In a study with 8141 singleton pregnancies, the PPV for T21, T18, and T13 was reported to be 80%, 60%, and 14.28%, respectively [27]; in another study with 42910 singleton pregnancies, the results were 79.23%, 54.84%, and 13.79%, respectively [28]. And the reported PPV range in several studies was 65-94% for T21, 47-85% for T18, and 12-62% for T13 [29–31]. Our results of PPVs for T21, T18, and T13 are within the range of these studies.

With the novel graphic-aided algorithm, the overall PPV was significantly increased to be 88.14% (*p* = 0.0041), and the PPV for T21 was also significantly increased from 71.43% to 93.02% (*p* = 0.0037). The PPV for T18, 88.89%, and T13, 57.14%, were also increased compared with the nNIPT methods, though the increases were not statistically significant. Compared with the reported PPVs, our gNIPT method has an advantage in lower false-positive cases, as it has a higher PPV for T21, T18, and T13.

The statistic *Z*-test utilized in the nNIPT treats a whole chromosome as one unit, and it could not properly handle situations where loss of regions and gain of regions in a chromosome offset each other. Samples have normal *z*-scores for each chromosome that may still have abnormity across the whole chromosome, with some regions having copy-number losses while other regions have copy-number gains. The gNIPT method in our study splits up each chromosome into a 2 Mb size window and treats each window as a unit for *z*-score calculation and CNV evaluation. It could detect small pathogenic CNVs as well as large regions of CNVs that affect the whole chromosome. With this gNIPT method, whole chromosomal aberration with chr18 was detected, in which a region of the chromosome was with onefold loss and another region was with threefold gain rendering the *z*-score of chr18 appeared to be within the normal range and could not be detected by nNIPT. In our study, gNIPT had detected one pathogenic CNV on chr17, which demonstrates the capability of gNIPT in detecting microdeletion and microduplication in the chromosome.

Due to the great economic burden the society and family might encounter in case of false-negative results, the accuracy of the widely used NIPT analysis method with *z*-score still needs improvement. Several studies have been engaged in developing better algorithms to improve the accuracy of the NIPT. For example, Xu et al. applied a Bayesian method that leverages informative priors on the fetal fraction [32], and Yang et al. tried to use a support vector machine to improve the calling of NIPT [33]. Most methods base the current detection on prior test results, which may be interfered by the characteristics of previous samples. Moreover, the theoretical models are sophisticated and need careful study to be comprehended, which impedes the clinical implementation of these methods. The gNIPT method presented is applicable to the current workflow in the data analysis for clinical needs. Further research on this kind of efficient and simple method is still needed, so that continual improvement in the accuracy of NIPT could be achieved.

## Figures and Tables

**Table 1:** Demographic characteristics of 13537 Chinese women.

Maternal age (years)	No. of samples	Average (years)	Percentage (%)
16-20	150	19	1.10
21-25	2665	24	19.70
26-30	5480	28	40.47
31-35	3247	33	23.99
36-40	1888	37	13.95
≥41	107	42	0.79
Gestational age (weeks)	No. of samples	Average (weeks)	Percentage (%)
12-15	984	15	7.27
16-19	10566	18	78.05
20-23	1813	21	13.40
24-27	172	25	1.27
28-31	1	29	0.00
36-40	1	36	0.00

**Table 2:** Comparison between gNIPT and nNIPT.

		Sensitivity (%)	Specificity (%)	PPV (%)	NPV (%)
Overall	gNIPT	98.11	99.95	88.14	99.99
	nNIPT	96.23	99.82	68.00	99.99
T21	gNIPT	97.56	99.98	93.02	99.99
	nNIPT	97.56	99.88	71.43	99.99
T18	gNIPT	100.00	99.99	88.89	100.00
	nNIPT	87.50	99.97	63.64	99.99
T13	gNIPT	100.00	99.98	57.14	100.00
	nNIPT	100.00	99.97	50.00	100.00

**Table 3:** Correlation analysis of gNIPT and nNIPT.

		nNIPT		Total
		Positive	Negative	
gNIPT	Positive	53	2	55
	Negative	18	13464	13482
	Total	71	13466	13537

## Data Availability

The data used to support the findings of this study are available from the corresponding author upon request.
